# Overexpression of mouse prion protein in transgenic mice causes a non-transmissible spongiform encephalopathy

**DOI:** 10.1038/s41598-022-21608-3

**Published:** 2022-10-13

**Authors:** Graham S. Jackson, Jacqueline Linehan, Sebastian Brandner, Emmanuel A. Asante, Jonathan D. F. Wadsworth, John Collinge

**Affiliations:** 1 MRC Prion Unit at UCL, UCL Institute of Prion Diseases, Courtauld Building, 33 Cleveland Street, London, W1W 7FF UK; 2 Division of Neuropathology, Queen Square Institute of Neurology, London, WC1N 3BG UK

**Keywords:** Prion diseases, Animal disease models

## Abstract

Transgenic mice over-expressing human *PRNP* or murine *Prnp* transgenes on a mouse prion protein knockout background have made key contributions to the understanding of human prion diseases and have provided the basis for many of the fundamental advances in prion biology, including the first report of synthetic mammalian prions. In this regard, the prion paradigm is increasingly guiding the exploration of seeded protein misfolding in the pathogenesis of other neurodegenerative diseases. Here we report that a well-established and widely used line of such mice (Tg20 or tga20), which overexpress wild-type mouse prion protein, exhibit spontaneous aggregation and accumulation of misfolded prion protein in a strongly age-dependent manner, which is accompanied by focal spongiosis and occasional neuronal loss. In some cases a clinical syndrome developed with phenotypic features that closely resemble those seen in prion disease. However, passage of brain homogenate from affected, aged mice failed to transmit this syndrome when inoculated intracerebrally into further recipient animals. We conclude that overexpression of the wild-type mouse prion protein can cause an age-dependent protein misfolding disorder or proteinopathy that is not associated with the production of an infectious agent but can produce a phenotype closely similar to authentic prion disease.

## Introduction

Prions are unique pathogens, devoid of coding nucleic acid, which self-propagate by means of seeded protein polymerisation to cause lethal neurodegenerative diseases in mammals^1,2^. They are fibrillar assemblies of prion protein (PrP) composed of misfolded host-encoded cellular prion protein (PrP^C^), some of which acquire protease-resistance and are classically designated as PrP^Sc^^1–4^. It is increasingly recognised that similar seeding processes may be involved in Alzheimer’s disease and other degenerative conditions and worldwide effort is now being made to establish the precise role of prion-like mechanisms in human disease^2,5–9^.

Transgenic lines of mice over-expressing wild-type or mutant human PrP on a mouse PrP knockout background have made critical contributions to our understanding of sporadic and acquired human prion diseases^10–12^ and have enabled accurate modelling of inherited prion diseases^10,13–15^. Numerous studies have reported substantial transmission barriers when investigating the zoonotic potential of prion strains. Inoculation of Chronic Wasting Disease (CWD) prions into humanised transgenic mice elicits no detectable clinical prion disease or abnormal pathology in mice up to 781 following inoculation^16^. Similarly, typical and atypical sheep scrapie isolates fail to transmit to humanised transgenic mice with no detectable pathology in mice up to 673 days post infection^17^. The study of mice over extended periods of time is common when investigating transmission barriers. A transmission barrier between sporadic CJD and wild-type mice was confirmed by a lack of any clinical prion disease or any detectable pathology in recipient mice after extended incubation periods^18^ whereas the introduction of human *PRNP* into PrP^0/0^ mice renders them susceptible to CJD prions. Modelling of inherited prion disease mutations has used humanised transgenic mice in long term natural history studies and established that for some mutations there is no detectable disease or pathology even in mice of up to 955 days old^13^. Similarly, transgenic lines of mice over-expressing mouse PrP on a mouse PrP knockout background have provided the basis for many of the fundamental advances in understanding prions and are widely used for prion bioassay. The essential role of host PrP for prion propagation and pathogenesis is demonstrated by the fact that knockout mice lacking PrP expression (*Prnp*^*o/o*^ mice) are entirely resistant to prion infection^19,20^ and that reintroduction of PrP transgenes restores susceptibility to infection in a species-specific manner that allows reverse genetics approaches to studying structure–function relationships in PrP (for reviews see^10,11,21–25^). The effects of PrP ablation were studied by several groups in the hope of establishing a role for the prion protein in brain anatomy or development. Although no consistent or significant phenotypes were ascribed numerous studies established that aged PrP^0/0^ knock-out mice from the Zurich 1 line did not display any anatomical or histological anbormalities^19,26^ even in excess of 720 days of age^27^.Attempts to create synthetic prions from recombinant PrP has been a major focus for many research groups in recent years, and such a demonstration would be the final vindication of the protein-only hypothesis of prion replication. Efforts have focused on the identification of conditions which modify the conformation of recombinant PrP and facilitate self-assembly into fibrillar states rich in beta-sheet structure. The bioassay host for such attempts is typically a transgenic mouse line selected for high levels of overexpression of PrP^C^ such as Tg20^28^ or Tg9949^29^ as high levels of PrP^C^ shorten the incubation period for prion disease and hence render the host animals able to report low titres of prion infectivity within their lifespan. This approach resulted in the first report of synthetic mammalian prions being assayed and replicated in transgenic mice^29^. Although there have been reports of prion synthesis resulting in the infection of wild-type rodents^30–32^, the use of transgenic mice engineered to overexpress a suitable substrate PrP remains widespread. In this regard, the prion paradigm is increasingly guiding the exploration of seeded protein misfolding in the pathogenesis of other neurodegenerative diseases^2,5–7^.

Despite the general utility of transgenic mice over-expressing wild type PrP in prion research some lines of mice have been found to develop spontaneous neurological dysfunction^33–36^. Studies of other neurodegenerative diseases must therefore recognise the fact that overexpression of a wild-type transgene may have deleterious effects and may result in the spontaneous formation of protein aggregates or proteinopathy in the absence of challenge with exogenous proteopathic seeds.

Here we describe the effects of high levels of overexpression of murine PrP in the commonly used Tg20 mouse line^28^, which express PrP at approximately eight-fold wild-type levels^37,38^ Animals inoculated with sterile phosphate buffered saline as control groups for other long term observational experiments were occasionally found to develop a clinical syndrome at times approaching their natural lifespan. This clinical syndrome was phenotypically similar to prion disease and neuropathological examination showed focal spongiform changes with abnormal PrP deposition and occasional neuronal loss. However, no protease resistant PrP could be detected by western blotting and affected brain homogenate failed to transmit the syndrome after re-inoculation into further groups of mice. We conclude that overexpression of wild-type mouse PrP can lead to a proteinopathy and the development of a non-transmissible encephalopathy which can confound attempts to assay for synthetic prions. This finding has wider relevance to studies of a range of other protein misfolding disorders where ‘prion-like’ transmission experiments utilise transgenic mice overexpressing wild type substrate proteins and potential transmission is established by the deposition of aggregated protein, in the absence of overt clinical disease. Whilst such models can be valuable for the study of ‘seeding’ activity associated with proteinopathies it is crucial to characterise the time course of potential spontaneous protein aggregation and associated pathology to distinguish between specific, seeded events and those arising merely from the over-expression of substrate.

## Results

### Histological findings in ageing Tg20 mice

Animals inoculated with either sterile Dulbecco’s phosphate-buffered saline lacking Ca^2+^ and Mg^2+^ ions (D-PBS) or sterile buffer (see Methods) showed no sign of a clinical syndrome within the usual latency period for the onset of prion disease, which in Tg20 mice is around 120 days for the transmission of RML at end-point dilution. However, continued long-term observation of animals to their natural lifespan resulted in a proportion of animals displaying clinical symptoms consistent with prion disease (n = 6/54, Table 1). In total the brains of 54 mice were examined, with ages ranging from 372 to 960 days at death. Following histopathological examination 25 out of the 54 animals examined were found to have abnormal pathology suggestive of prion disease including all six mice that had clinical signs of prion disease.

**Table 1:** Incidence of clinical signs and neuropathological changes in Tg20 mice challenged with D-PBS or buffer.

^a^Inoculum	^b^Clinically affected/Inoculated	^c^Survival Time, mean ± SEM days	^d^Neuropathology Positive/Inoculated
Sterile PBS	4/24	691 ± 14	9/24
Sterile buffer	2/30	666, 705	16/30

^a^Groups of mice were inoculated intracerebrally with either D-PBS as a standard inoculation control or with sterile buffer comprising 20 mM Tris + 20 mM sodium acetate + 200 mM NaCl pH 4.0. Mice were then observed daily over their lifespan and culled if showing signs of clinical prion disease, distress caused by inter-current illness, or senescence or at termination of the experiment.

^b^Clinical disease was defined by the criteria described in Methods.

^c^Survival times are reported for mice with clinical signs consistent with prion disease in days post inoculation; where n ≥ 3 the mean ± SEM is reported, otherwise individual survival times are given.

^d^Brains from all 54 mice reported in the Table were examined and classified by neuropathological examination and immunohistochemistry. Positive neuropathology comprises spongiform change, either alone, or in combination with the presence of abnormal PrP deposition.

Sections were stained with haematoxylin and eosin (H&E) as well as immunostaining for glial fibrillary acidic protein (GFAP) and PrP. The brain regions examined included cortex, hippocampus, striatum/basal ganglia, thalamus, brain stem, cerebellum, and all white matter tracts. A total of 29 mice lacked any unusual or pathological features, whilst the remaining cohort of 25/54 displayed spongiform degeneration, which in some mice was associated with deposition of abnormal PrP. An additional variable feature of cerebellar degeneration was also observed in a proportion of the animals.

**Figure 1:**
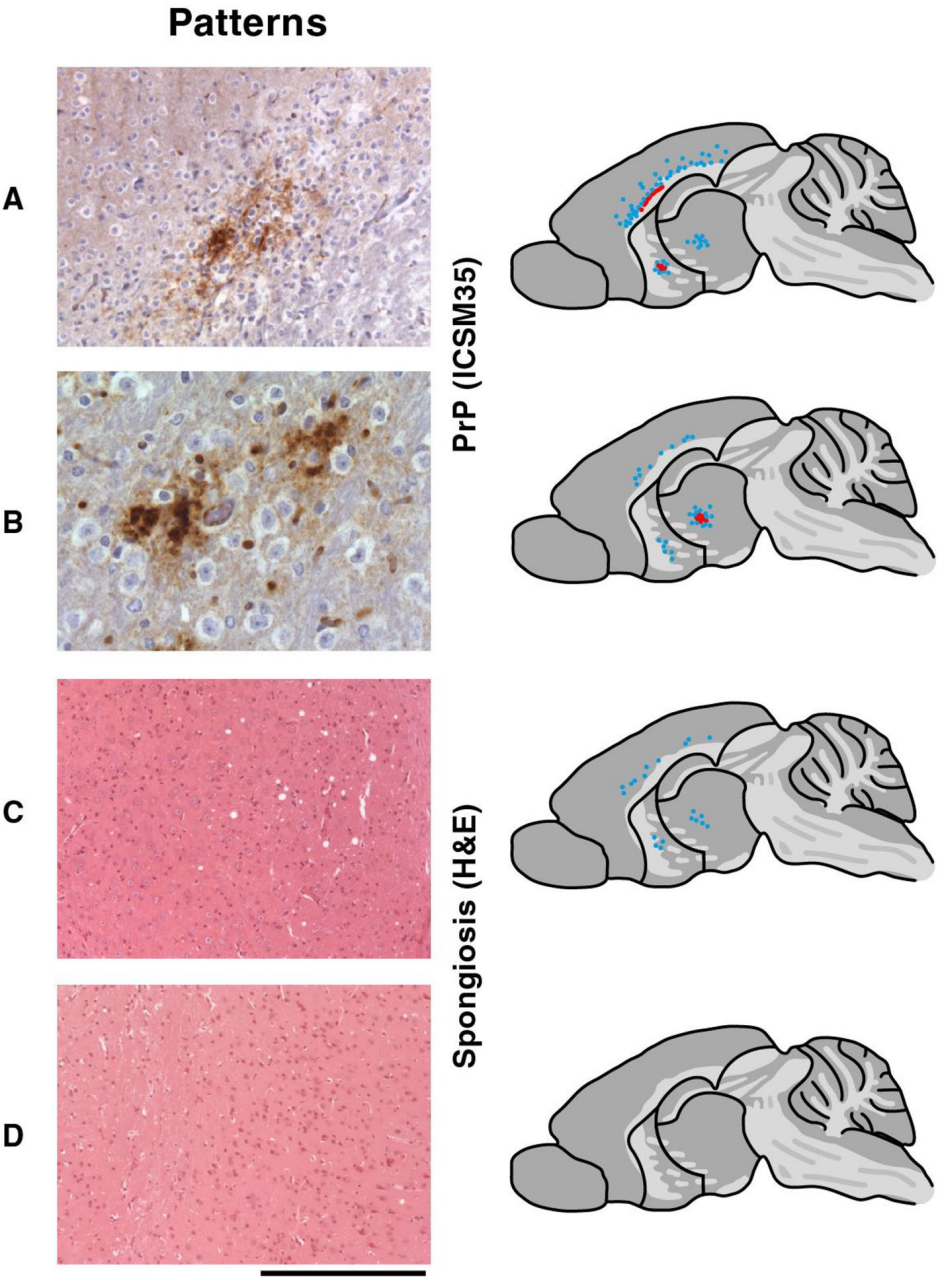
Distribution of spontaneous abnormal PrP and spongiform change in the brain of aged Tg20 mice challenged with D-PBS or buffer: The left hand panels show the typical histology which is the predominant feature of each group. (**A** and **B**) PrP immunohistochemistry using anti-PrP monoclonal antibody ICSM35 revealed abnormal PrP deposition including PrP-positive plaques. (**C** and **D**) Haematoxylin and eosin (H&E) stained sections showing spongiform neurodegeneration when present. Scale bar: 150 µm (**A**), 75 µm (**B**), and 300 µm (**C** and **D**). The right hand panels show schematic representations of mouse brain and indicate the spatial distribution of the pathology typical for each group. Abnormal PrP deposition is indicated in red and spongiform degeneration in blue.

The deposition of abnormal PrP was not consistent in all brains examined but was observed in two distinct locations. One group of mice (n = 8, Fig. 1, Pattern A) showed small subcortical deposits of abnormally aggregated PrP, which was partly of a synaptic pattern, but focally condensed to a granular pattern and very occasionally formed microplaques. These deposits were predominantly in the deep cortical layers adjacent to the corpus callosum. They extended in the anterior–posterior direction as well as medio-laterally. The basal ganglia and thalamus were also occasionally affected, but to a much lesser extent. In the deep cortical/subcortical areas the PrP deposits were associated with spongiform degeneration of neurones, resulting in shrinkage of the deep cortical layers and the corpus callosum with an accompanying gliotic reaction compared to the group with no abnormal features or pathology. Spongiform degeneration and gliosis were typically more widespread than the corresponding PrP deposition (see Fig. 1).

The other major pattern of PrP deposition was a discrete small area restricted to the thalamus (n = 5, Fig. 1, Pattern B). Here the PrP deposits were synaptic and only rarely formed minute plaques. This was closely associated with a spongiform degeneration with some neuronal loss and considerable gliosis, which extended over a larger area in the thalamus. A variation of this second pattern was spongiform degeneration in the same thalamic area, with accompanying gliosis but a lack of PrP deposition (n = 12, Fig. 1, Pattern C). Although this pattern can be regarded as a separate group, it may reflect a less progressed disease stage in which abnormal PrP has not been deposited to a detectable level.

The additional pathological feature in all groups with spongiosis or spongiosis coupled with PrP deposition (i.e. all mice except group D) was a moderate atrophy of the cerebellar granule layer (n = 8/25). The monoclonal antibody ICSM35 that was used for PrP immunohistochemical staining has been widely used and does not cross-react with any non-specific targets in either wild-type and transgenic mice^17,39–41^.

The majority of animals comprised a fourth group (n = 29, Fig. 1, Pattern D) in which no pathology was detectable.

**Figure 2:**
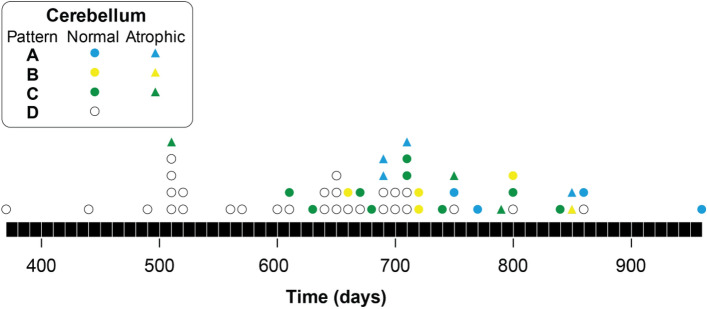
The occurrence of spontaneous neuropathology in ageing Tg20 mice challenged with D-PBS or buffer: The x-axis represents the latency in days from inoculation to death with each symbol representing an individual animal (see Table 1). Triangles represent mice with cerebellar atrophy and circles indicate no atrophy. Blue symbols represent pattern (**A**), yellow symbols pattern (**B**), green symbols pattern (**C**) and (**D**) with open circles representing unaffected animals with no signs of abnormal pathology (see Fig. 1).

**Figure 3:**
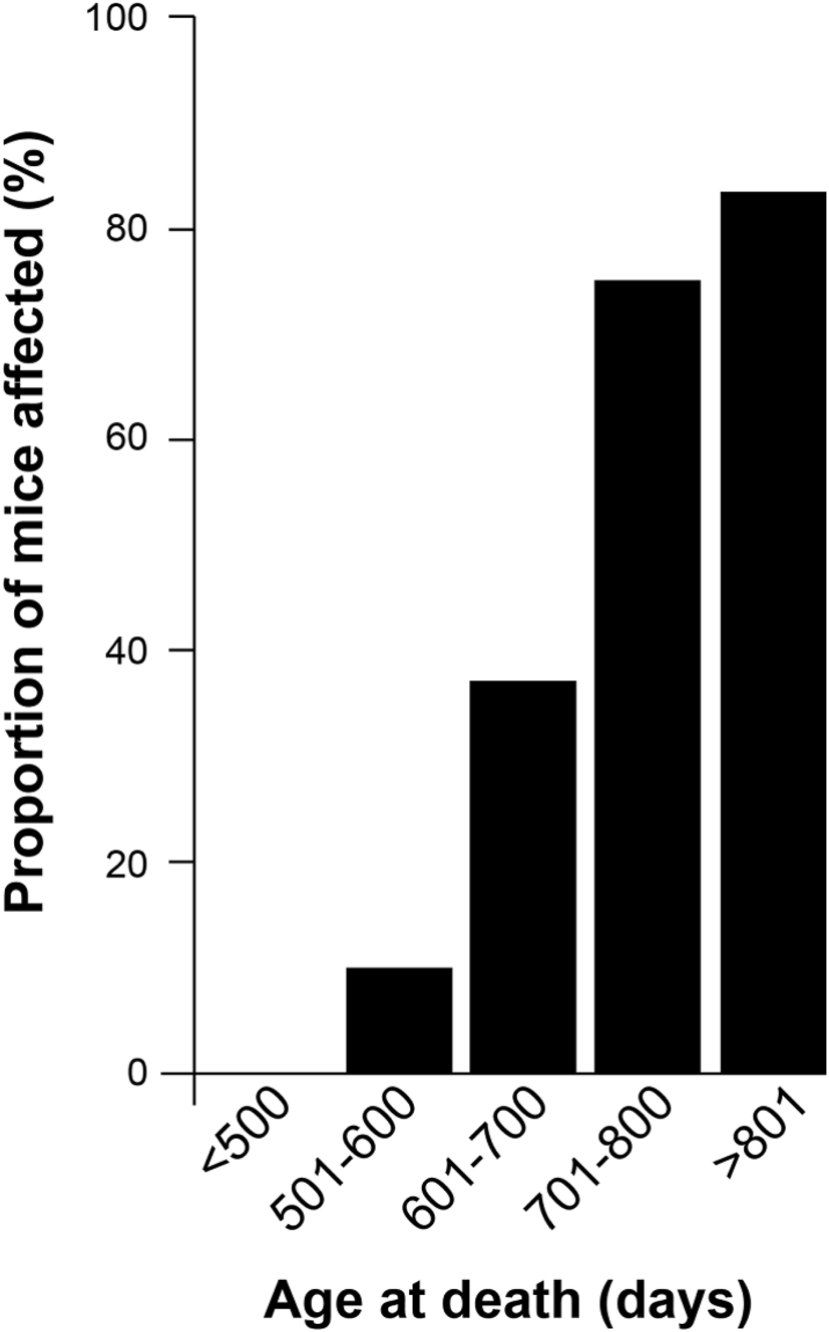
Age dependence of spontaneous neuropathology in Tg20 mice challenged with D-PBS or buffer. The proportion of Tg20 mice affected by neuropathological changes of all types (with patterns **A**, **B** and **C**) are shown as a percentage of the total number of mice examined in each of the age cohorts indicated on the x-axis. The numbers of mice affected were; < 500 days 0/3, 501–600 days 1/10, 601–700 days 7/18, 701–800 days 12/17 and > 801 days 5/6.

The development of spontaneous pathology and disease was strongly age-dependent with no animals affected within the normal time frame for prion transmissions (approximately 120 days post-inoculation (dpi)) and up to 500 days of age. Beyond 500 days an increasing proportion of animals were affected (Figs. 2 & 3), with over 80% displaying signs of spontaneous disease at ages approaching the natural lifespan of the animals. The mean age at death for animals unaffected was 614 days (SEM ± 20) compared to 741 days (SEM ± 18) for animals with pathology; a difference which is highly significant (*p* < 0.0001, two-tailed unpaired t test). This is a counter-intuitive observation for which there is no obvious explanation which further highlights the potential problems of interpreting results from highly aged transgenic animals.

### Second passage of brain homogenate from affected and unaffected mice

To establish if clinical signs and abnormal pathology were the result of a transmissible spongiform encephalopathy, brain homogenates were prepared from affected and unaffected mice and used to inoculate further experimental animals. Brain was inoculated intracerebrally as 1% w/v homogenate in D-PBS into Tg20 and animals were again monitored throughout their natural lifespan for signs of clinical prion disease. As in the cohort of Tg20 animals that received D-PBS or buffer inocula, a proportion of Tg20 recipients again developed a clinical syndrome (Table 2). However, the proportion of animals that were observed to be affected did not increase significantly from the frequency observed in the primary inoculation cohort challenged with either D-PBS (4/24 vs. 1/14 and 11/48; *p* = 0.63 and 0.76 respectively), or buffer (2/30 vs. 3/19 and 11/45; *p* = 0.36 and 0.063 respectively). Although we observed a slight reduction in the apparent incubation periods for the onset of clinical signs in these secondary transmissions (598 days and 552 days) (Table 2) compared to the primary inoculation cohort (691 and 686 days) (Table 1), this was modest and is not consistent with reductions of incubation period that would be associated with the transmission of authentic prions.

**Table 2:** Incidence of clinical signs and neuropathological changes following second passage into further Tg20 mice.

^a^Inoculum and classification	^b^Clinically Affected/Inoculated	^c^Incubation time, mean ± SEM (days)	^d^Neuropathology Positive/Inoculated
PBS unaffected mouse	0/5	–	2/4
PBS unaffected mouse	0/5	–	1/4
PBS unaffected mouse	1/4	538	2/4
Total	1/14	538	5/12
PBS affected mouse (NP pattern A)	0/13	–	9/12
PBS affected mouse (NP pattern A)	2/10	600, 638	5/9
PBS affected mouse (C,NP pattern B)	4/15	596 ± 33	6/11
PBS affected mouse (C,NP pattern B)	5/10	600 ± 3.8	4/6
Total	11/48	598 ± 13	24/38
Buffer unaffected mouse	0/5	–	1/3
Buffer unaffected mouse	3/9	567 ± 14	4/5
Buffer unaffected mouse	0/5	–	2/3
Total	3/19	567 ± 14	7/11
Buffer affected mouse (NP pattern A)	3/15	597 ± 17	10/13
Buffer affected mouse (C,NP pattern A)	4/15	531 ± 49	8/12
Buffer affected mouse (NP pattern B)	0/5	–	2/3
Buffer affected mouse (NP pattern A)	4/10	540 ± 59	1/2
Total	11/45	552 ± 27	21/30

^a^Inocula comprised 1% w/v brain homogenate from Tg20 mice originally challenged intracerebrally with D-PBS or Buffer (Table 1). Unaffected mice showed no clinical signs consistent with prion disease and no neuropathological changes whereas affected mice were either positive for clinical signs consistent with prion disease (C) or positive for neuropathological changes (NP) or both (C, NP). Inoculated mice were observed daily over their lifespan and culled if showing signs of clinical prion disease, distress caused by inter-current illness, or senescence or at termination of the experiment.

^b^Clinical disease was defined by the criteria described in Methods.

^c^Survival times are reported for clinically affected mice in days post inoculation; where n ≥ 3 the mean ± SEM is reported otherwise individual incubation times are given.

^d^Positive neuropathology comprises spongiform change, either alone, or in combination with the presence of abnormal PrP detected by immunohistochemistry.

Neuropathological features were also observed in a proportion of the second passage animals receiving homogenised brain tissue. Again compared to the primary inoculation cohort there was no significant increase in the frequency of these features (D-PBS 9/24 vs. 5/12 and 24/38; *p* = 1.0 and 0.068 respectively or Buffer 16/30 vs. 7/11 and 21/30; *p* = 0.73 and 0.29 respectively), indicating that their origin is due to a spontaneous event.

### Western blot detection of protease resistant PrP in the brains of affected mice

None of the animals tested from either the original primary inoculation group or second passage groups contained any protease-resistant PrP that could be detected by western blotting, either directly, or after sodium phosphotungstic acid precipitation^42,43^ of 0.25 ml 10% w/v brain homogenate.

## Discussion

Enormous advances in our understanding of prion diseases are now providing a paradigm for other human diseases involving the accumulation of misfolded host proteins. Elucidating the mechanisms that govern the formation, transmissibility and toxicity of misfolded protein seeds in other neurodegenerative diseases is now a major focus of worldwide research^2,5–8^. Key to the advances made in prion biology has been the development of highly specialised tools and animal models to explore transmissibility and pathogenesis. Animal models of prion disease have been extensively studied and authentically reproduce the pathology seen in human patients, whilst isolation of the infectious agent has yielded structural insights that differentiate prions from other forms of amyloid^3,4,44^.

Although the development of seminal methods for prion research took decades to establish, rapid advances in other diseases involving proteopathic seeds can be envisaged using the research approaches and strategic framework that has been established in prion disease. Over the next few years it is likely many new transgenic models will be developed to study a wide range of neurodegenerative diseases using experience from the prion field for critical guidance.

While PrP is very well known to be capable of misfolding to produce infectious prions, our findings in aged Tg20 mice now show that PrP can also misfold and aggregate to produce a non-transmissible proteinopathy. The overt neuropathological features of Tg20 mice affected by this proteinopathy are closely similar to those that characterise prion disease; comprising accumulation of abnormal PrP, spongiform degeneration and neuronal loss. In some cases, although not all, pathology can be associated with a clinical syndrome that mirrors classical prion disease in these mice. Although it formally remains a possibility that the site of transgene insertion could be responsible for these effects, our results indicate that accumulation of abnormal PrP is likely to at least contribute to the process responsible for the vacuolation and neuronal loss associated with prion infection that in turn may lead to a clinical disease when sufficiently advanced. Since we saw no evidence for the generation of transmissible prions in the brain of any clinically affected or unaffected mouse, our findings suggest specific processes are required to form infectious prions distinct from the propagation of PrP amyloid^9^.

A previous study explored the possibility that overexpression of *Prnp* in Tg20 mice might lead to spontaneous prion formation^45^. Serial passages of brain homogenate obtained from Tg20 mice were carried out for three generations with each generation being observed for 250 days before sacrifice and histological examination. The authors did not observe any clinical disease or abnormal neuropathology in these mice which is consistent with our observations that the earliest occurrence of proteinopathy had a latency of over 500 days (Table 2, Figs. 2 and 3). Cognisant of protein aggregation being a time-dependent process the authors also conducted long-term observations of six Tg20 mice inoculated with brain homogenate obtained from aged Tg20 animals. Of the six recipients, three developed a neurological syndrome with associated misfolded PrP accumulation in the brain with a mean incubation period of 422 days. The authors suggest this is consistent with low levels of prion infectivity (~ 1ID_50_/3 mg tissue) in the original brain used to prepare the inoculum^45^. However they did not passage brain material from the affected mice to confirm the presence of transmissible prions and our results provide an alternative explanation for their findings.

The use of sub-passage is extremely useful for confirming the presence of prion infectivity. Hence, where we observed signs of disease, brain homogenates were passaged to further groups of mice. We recorded a similar pattern of phenotypes in recipients with no significant increase in the number of animals affected. Third passage can be useful in some circumstances and has been used to assay low concentrations of prions^46^. However, we did not perform a third passage as there was no evidence of any prion transmissions or amplification of prions by second passage. Although we observed a slight reduction in apparent incubation period for onset of clinical signs in these secondary transmissions (Table 2) the extent of adaptation is not congruent with transmission of authentic prions. The observation of a clinical syndrome and neuropathology reminiscent of prion disease in aged Tg20 mice unexposed to exogenous prion inoculum is an important finding with respect to animal models suitable for studies aimed at generating high titre synthetic prions from recombinant PrP where long-term observation is required.

As the prion paradigm is applied to an ever widening group of protein misfolding disorders efforts are increasing to establish if other neurodegenerative diseases might be transmissible in humans^8,47^. Our results highlight the importance of fully characterising experimental models overexpressing wild type amyloidogenic substrate proteins and the need for caution when interpreting the results of long term observations approaching the natural lifespan of transgenic animals in the absence of suitably studied age-matched controls.

## Methods

### Inoculation of transgenic mice

All procedures were conducted in microbiological containment level 3 facilities with strict adherence to safety protocols. All experiments performed with animals were performed in accordance with licences approved and granted by the UK Home Office (Project Licences P7B5EB63F, 70/6454 and 70/7274) and conformed to University College London institutional and ARRIVE guidelines. All experimental protocols were approved by the Local Ethics Committee of UCL’s Institute of Neurology and National Hospital for Neurology and Neurosurgery. Inoculation of Tg20 mice^28^ was carried out according to strict biosafety protocols in a class I microbiological safety cabinet. All mice were inoculated with a total volume of 30 µl comprising either sterile Dulbecco’s phosphate-buffered saline lacking Ca^2+^ and Mg^2+^ ions (D-PBS) (Invitrogen), buffer (20 mM Tris 20 mM sodium acetate + 200 mM NaCl pH 4.0) or 1% w/v brain homogenate intra-cerebrally into the right parietal lobe following anaesthesia with halothane/O_2_.

### Animal husbandry and criteria for determining clinical disease

Animal husbandry was carried out as previously described^12^. Briefly, all mice were examined daily for early indicators of clinical prion disease including piloerection, sustained erect ears, intermittent generalised tremor, unsustained hunched posture, rigid tail, mild loss of coordination, and clasping hind legs when lifted by the tail. Definite diagnosis of clinical prion disease (triggering experimental end point) was reached if mice exhibited any two early indicator signs in addition to one confirmatory sign, or any two confirmatory signs. The confirmatory signs included ataxia, impairment of righting reflex, dragging of hind limbs, sustained hunched posture, or significant abnormal breathing^12,40,41^. Mice were killed (by CO_2_ asphyxiation) if they exhibited any signs of distress or once a diagnosis of prion disease was established. At post-mortem brains from inoculated mice were removed, divided sagittally with half frozen and half fixed in 10% buffered formol saline. Subsequent immunohistochemical or biochemical investigations were performed blind to sample provenance.

### Preparation of brain homogenates and western blot detection of PrP

Frozen brain, right hemispheres, were prepared as a 10% w/v homogenates in D-PBS using tissue grinders (Anachem) and dispensed into aliquots and stored at − 80 °C. For preparation of inocula aliquots of 10% w/v homogenates were thawed and diluted to 1% w/v in D-PBS and serially passaged through syringe needles of decreasing diameter. For analysis by immunoblotting 10% w/v homogenate was clarified by removing gross cellular debris by centrifugation at 1000 rpm (80 × g) for 1 min in a microfuge (Eppendorf). Detection of proteinase K-resistant PrP in the supernatant was performed with and without enrichment by sodium phosphotungstic acid precipitation as described previously^43,48^ using anti-PrP monoclonal antibody ICSM35 (D-Gen Ltd, London).

### Neuropathology and PrP immunohistochemistry

Left brain hemispheres fixed in 10% buffered formol saline were assessed for presence of abnormal PrP by staining with the anti-PrP monoclonal antibody ICSM35 (D-Gen, London) using a Ventana automated immunohistochemical staining machine (Ventana Medical Systems, Tuscon, AZ, USA) as described previously^48^.Tissue was fixed in 10% v/v buffered formol saline followed by incubation in 98% v/v formic acid for 1 h. Following further washing for 24 h in 10% v/v buffered formol saline tissue samples were processed and embedded in paraffin wax. Sections were cut at a nominal thickness of 4 µm, treated with 98% v/v formic acid for 5 min and the slides were placed on the automated staining machine. De-paraffinisation was performed with xylene followed by heating to 95 °C in a low ionic strength buffer (2.1 mM Tris, 1.3 mM EDTA, 1.1 mM sodium citrate, pH7.8) for 30 min, before 16 min protease treatment. Abnormal PrP accumulation was detected using anti-PrP monoclonal antibody ICSM35^49^ in conjunction with a biotinylated-anti-mouse IgG secondary antibody (*i*View SA-HRP, Ventana Medical Systems) before development with 3′3 diaminobenzidine tetrachloride as the chromogen (iView DAB, Ventana Medical Systems). Immunostaining for glial fibrillary acidic protein (GFAP) and haematoxylin and eosin (H&E) staining of serial sections was done according to published methods^48^.

### Ethics approval

Work with animals was performed in accordance with licences approved and granted by the UK Home Office (Project Licences PPL: P7B5EB63F, 70/6454 and 70/7274) and conformed to institutional and ARRIVE guidelines.

## Data Availability

The datasets used and analysed for this study are available from the corresponding author on reasonable request.
